# Comparative Analyses of Liquid-Biopsy MicroRNA371a-3p Isolation Protocols for Serum and Plasma

**DOI:** 10.3390/cancers13174260

**Published:** 2021-08-24

**Authors:** Dennis M. Timmerman, Ad J. M. Gillis, Michal Mego, Leendert H. J. Looijenga

**Affiliations:** 1 Princess Máxima Center for Pediatric Oncology, 3584 CS Utrecht, The Netherlands; D.M.Timmerman-6@prinsesmaximacentrum.nl (D.M.T.); A.J.M.Gillis@prinsesmaximacentrum.nl (A.J.M.G.); 2 Translational Research Unit and 2nd Department of Oncology, Faculty of Medicine, Comenius University and National Cancer Institute, 84505 Bratislava, Slovakia; misomego@gmail.com

**Keywords:** cancer, clinical investigation, molecular diagnostics, real-time PCR, quantitative analysis of nucleic acids

## Abstract

**Simple Summary:**

The active disease status of patients with a malignant germ cell tumor can be evaluated using detection of specific body-circulating microRNAs. However, various methods are reported to isolate and detect microRNAs from blood, possibly influencing the score as positive or negative. Here, we investigated two frequently used techniques for microRNA isolation from blood, either serum or plasma, to evaluate possible differences. These data are required to compare published studies and to select the best methods in the future. No effect of either starting with plasma or serum was found, indicating that both blood products can be used. The bead-based method was more stable and applicable on small blood volumes, whereas the total RNA method exhibited a higher sensitivity due to a larger starting volume. These results are important to develop the optimal method for the detection of microRNAs in blood to monitor malignant germ cell tumor patients in clinic practice.

**Abstract:**

MicroRNAs (miRNAs) are short, non-coding RNAs involved in translation regulation. Dysregulation has been identified in cancer cells. miRNAs can be secreted and detectable in body fluids; therefore, they are potential liquid-biopsy biomarkers. The miR-371a-3 cluster members are an example, monitoring the presence of malignant germ cell tumors based on patient serum/plasma analyses. However, a large variety of isolation techniques on sample types (serum vs. plasma) are reported, hampering interstudy comparisons. Therefore, we analyzed the impact of using the miRNeasy Serum/Plasma Kit (cell-free total RNA purification) Qiagen extraction kit and the TaqMan anti-miRNA bead-capture procedure of ThermoFisher for miRNA isolation. Ten normal male matched serum and plasma samples and seventeen testicular germ cell tumor patient serum samples were investigated. The Qiagen kit requires a higher input volume (200 µL vs. 50 µL), resulting in higher sensitivity. Serum and plasma comparison demonstrated high similarity in miRNA levels. Titration experiments showed that the bead-capture procedure is superior in cases of lower starting volumes (<100 µL). This study highlights the strengths and limitations of two different isolation protocols, relevant for in vivo analysis with small starting volumes. In summary, miRNA detection levels results varied little between plasma and serum, whereas for low volumes the bead capture isolation method is preferable.

## 1. Introduction

Biomarkers are known to be a strong clinical tool to detect, diagnose and stratify cancer patients as well as aid in the development of new treatments by predicting patient responses and outcomes [1]. In this context, microRNAs (miRNAs) are potentially highly useful as molecular biomarkers because they can be disease-specific, are often secreted into bodily fluids, are stable with a short half-life, and are relatively easy to extract and detect [2]. Secreted miRNAs are therefore extremely interesting as clinical liquid-biopsy biomarkers because they can mark the cells of (tumor) origin and can be used as clinical tools for disease monitoring (e.g., whole blood, serum or plasma and cerebrospinal fluid) [3]. However, various isolation and determination techniques are characterized by different sensitivity and specificities; therefore, determining a precise cut-off between the different methods has been challenging the field of liquid-biopsy-based biomarker applications [4,5].

Liquid-biopsy-based miRNA biomarkers have, for example, been demonstrated to be particularly useful in (testicular) germ cell tumors ((T)GCTs) [6,7,8,9,10,11]. The levels of the miRNA cluster 371a-373 (normally specifically present during embryonal development) are elevated in 87% in seminomas and in more than 90% of non-seminomatous (T)GCT patients (teratomas excluded), while hardly detected in healthy individuals (11), excluding false positive findings. However, the detection limit, precision and specificity of various extraction methods and isolation protocols have not yet been compared extensively, thereby increasing the risk of identifying false positive and negative cases. We exploit the already proven high specificity and sensitivity of hsa-miRNA371a and 373 to demonstrate a clean comparison between isolation protocols and starting material [11].

To shed light on these aspects, we performed a relatively simple, although highly informative, comparative study using matched serum and plasma samples from 10 healthy male donors (age 18–40 years) and 17 serum samples from diagnosed TGCT patients. The miRNeasy Serum/Plasma Advanced Kit Qiagen extraction kit (cell-free total RNA purification) was compared to the TaqMan anti-miRNA bead-capture procedure (~370 miRNAs) from ThermoFisher. This comparison was specifically investigated because of the various methods used in the field possibly relating to variations in results [7,8,12,13,14] or conditions [4,5,15,16,17,18,19,20,21]. Together with the use of various isolation methods, differences between serum and plasma samples have been suggested to explain results between studies [8,12,13,14,18]. Our results demonstrate a lower detection limit for the cell-free total RNA purification of the Qiagen kit compared to the bead-based method, whereby the latter generally showed a lower variation in isolation efficiency. Furthermore, using hsa-miR371a-3p, we demonstrate that the bead-based method is more sensitive to low levels of this specific miR target. We conclude that both serum and plasma samples can be used as liquid-biopsy starting material to detect hsa-miR371a-3p as molecular biomarker for TGCTs, whereas the Qiagen kit generally has a lower detection limit in exchange for lower precision. Furthermore, the bead-capture procedure is superior even in cases of small starting voluminal amounts of the sample. These data are relevant in the context of development of the most stable, sensitive and specific method for final clinical applications of hsa-miR371a-3p in liquid biopsies. 

## 2. Materials and Methods

### 2.1. Patient and Control Serum/Plasma Samples

Use of patient samples remaining after diagnosis was approved for research by the Medical Ethical Committee of the EMC (The Netherlands), permit no. 02.981. This included permission to use the secondary samples without further consent. Samples were used according to the “Code for Proper Secondary Use of Human Tissue in The Netherlands” developed by the Dutch Federation of Medical Scientific Societies (FMWV, version, 2002; update 2011). The use of patient samples provided by Dr. Michal Mego was approved according to institutional board review (2020). This retrospective translational study was approved by the Institutional Review Board (IRB) of the National Cancer Institute.

### 2.2. MiRNA Purification

miRNAs were isolated from 50 µL serum and plasma using target-specific anti-miR magnetic beads, as reported before (OncoTarget 2016). In short, a KingFisher Flex robot with TaqMan® miRNA ABC Purification Kit Human Panel A (ThermoFisher PN 4473087, Waltham, MA, USA) was used to isolate miRNAs. All reagents are provided in the kit. These panels consist of superparamagnetic Dynabeads covalently bound to a unique set of ~380 anti-miR oligonucleotides. Briefly, 100 µL of lysis buffer (containing spike-in) was added to 50 µL of serum/plasma, followed by the addition of 80 µL of beads (10^6^ beads/µL). Samples were incubated at 30 °C for 40 min, then washed three times with wash buffer. The bound miRNAs were eluted from the beads with 100 µL elution buffer.

RNA from 200 µL of thawed serum and plasma was isolated using the miRNeasy Serum/Plasma Advanced Kit from Qiagen (PN 217204), according to the manufacturer’s instructions. In order to increase RNA yield, MS2 carrier RNA (Roche PN 10165948001) was added to a final concentration of 1.25 µg/mL. Total RNA was eluted from columns with 50 µL of nuclease-free water. During the lyses of the samples, a non-human spike-in Cel-miR39 (5.6 × 10^8^ copies) external control was added to each sample, in both isolation-techniques, to monitor the RNA recovery.

### 2.3. Quality Control Assessment

To check the RNA recovery and suitability for use in subsequent RT-PCR, 1 µL of the purified miRNA/RNA was reverse-transcribed using a TaqMan miRNA RT Kit (PN 4366597) and TaqMan miRNA assays for Cel-miR39-3p (000200) and hsa-miR30b-5p (000602). miRNA levels were detected on a QuantStudio 12K Flex machine. 

### 2.4. Hemolysis Assessment

Hemolysis levels were evaluated according to the “miR-32a/451a” ratio, as previously reported [22]. Furthermore, it was assessed by visual inspection as previously reported by Lobo et al. [7]. No samples were discarded after both assessments.

### 2.5. Target-Specific Real-Time PCR

For miRNA profiling, 5 µL of the purified miRNA/RNA was reverse-transcribed using TaqMan miRNA RT Kit (PN 4366597) and an equal mixture of the RT-primers of Cel-miR39-3p (000200), hsa-miR30b-5p (000602), hsa-miR371a-3p (002124), hsa-miR373-3p (000561), and hsa-miR375 (000564). The final volume of 15 µL for each reaction underwent RT using a BioRad T100 Thermal Cycler at 16 °C for 30 min, 42 °C for 30 min, followed by a final step of 85 °C for 5 min. To increase sensitivity and specificity, a 12-cycle pre-amplification step was included. Briefly, an equal mix of all 20× TaqMan miRNA assay probes was prepared for each reaction and diluted to 0.2× with 1× Tris-EDTA Buffer (pH 8.0). Each sample contained 12.5 µL 2× TaqMan PreAmp Master Mix (PN 4488593), 7.5 µL of diluted TaqMan assay probe mix, and 5 µL of multiplexed cDNA product. After heating to 95 °C for 10 min, 12 cycles of 95 °C for 15 s and 60 °C for 4 min were run on a thermal cycler (BioRad). The resulting reaction products were diluted 1:4 with nuclease-free water to a final volume of 100 µL. For the final singleplex PCR, 1.5 µL of the diluted pre-amplification product was added to 10 µL 2× TaqMan Advanced PCR Master Mix (PN 4444964), and 1 µL of each individual 20× TaqMan primer/probe assay. All reactions were performed in duplicate. miRNA levels were determined on a QuantStudio 12K Flex machine. All kits were purchased from Thermo Fisher Scientific, Bleiswijk, The Netherlands.

### 2.6. Data Normalization and Analysis

For normalization, endogenous reference hsa-miR30b-5p was used as described previously [23]. miRNA levels were relatively quantified according to the 2−ΔΔCt method after normalization to housekeeping hsa-miR30b-5p. Targets were corrected for hsa-miR30b-5p values corrected for average hsa-miR30b-5p levels in the total population to correct for deviations in the endogenous levels of hsa-miR30b-5p. Data were processed using Excel, and data were visualized using GraphPad Prism 9.3. Statistical significance was determined using an unpaired two-tailed Student’s *t*-test.

## 3. Results

### 3.1. Sample Preparation and Quality Control

**Figure 1:**
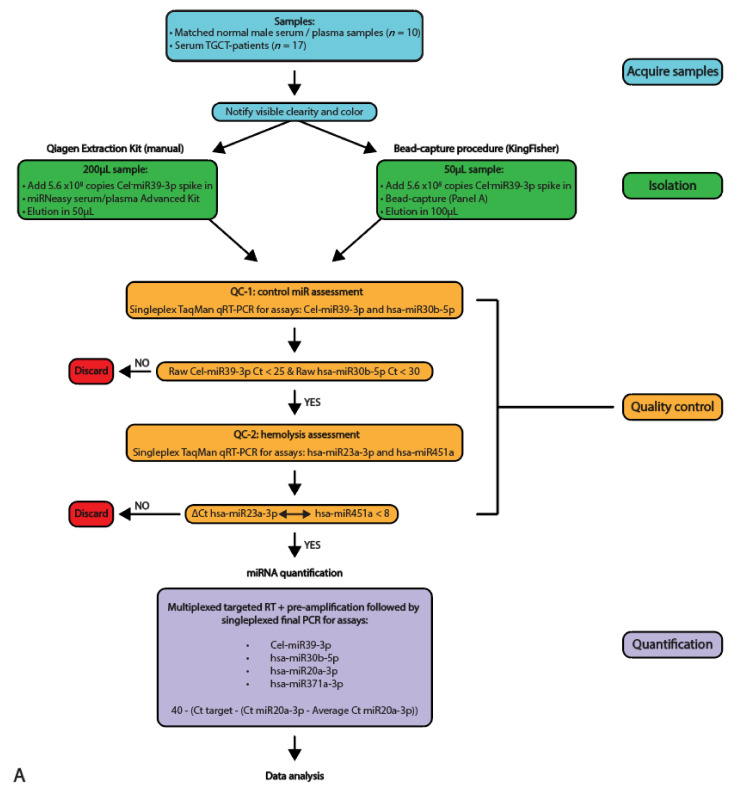

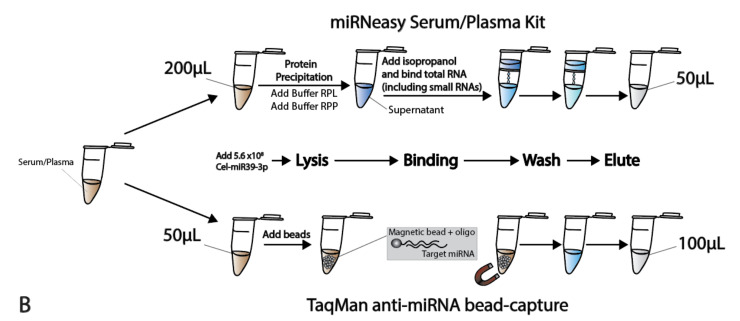
Schematic overview of the study set-up and workflow. (**A**) A schematic overview of the study setup used, depicting the acquiring of the samples, isolation, quality control and quantification techniques used. (**B**) Schematic overview of the workflow used with the two isolation protocols (Qiagen kit and bead-capture).

**Figure 2:**
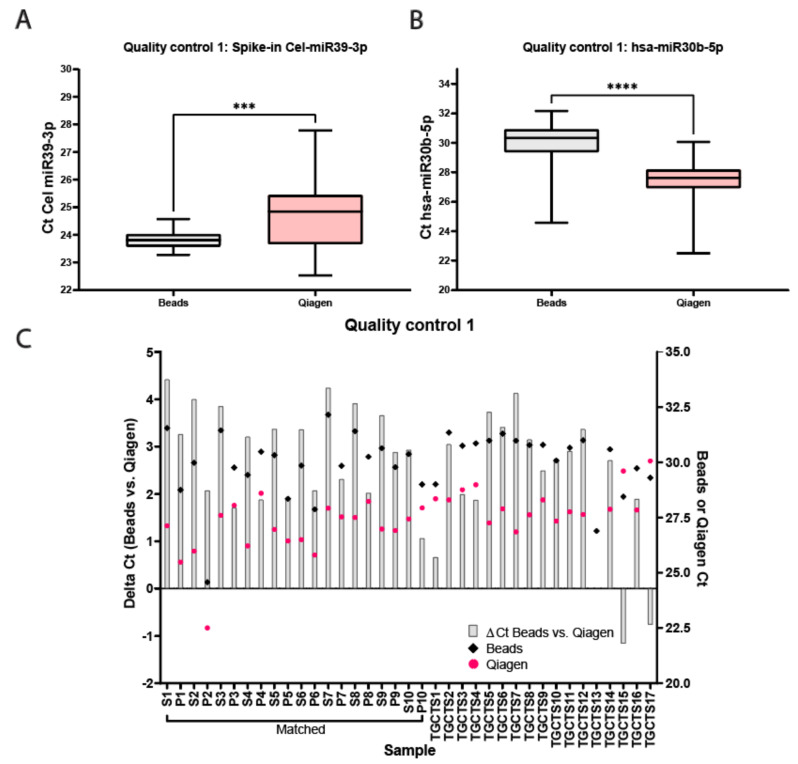
Quality control for bead-capture and Qiagen kit isolation. (**A**) Cts for Cel-miR39-3p spike-in quality control isolated with beads (black) or Qiagen (pink). Average Ct Beads: 23.81 ± 0.33, Average Ct Qiagen 24.68 ± 1.32, *** *p* < 0.005. (**B**) Cts for hsa-miR30b-5p endogenous quality control isolated with beads (black) or Qiagen (pink). Average Ct Beads: 29.98 ± 1.45, Average Ct Qiagen 27.45 ± 1.30, **** *p* < 0.0001. (**C**) Delta Cts of hsa-miR30b-5p between the bead-capture and Qiagen kit isolation (grey bars). Raw Cts obtained with the bead (black) of Qiagen (pink) isolation protocol are plotted on the right *y*-axis.

miRNAs from 10 unrelated matched serum and plasma samples originating from control males and 17 serum samples from independent TGCT patients were isolated using the two different methods and qualitatively analyzed and quantified using qRT-PCR (full study set-up and workflow are displayed in Figure 1A,B, sample names are presented in Table 1). The samples were first subjected to multiple quality control experiments before being analyzed quantitively for miRNA levels. Figure 2A,B display box-plotted Ct values for the spike-in Cel-miR39-3p (Median Ct Beads: 23.81 ± 1.3, Median Ct Qiagen 28.84 ± 5.25) and endogenous hsa-miR30b-5p (Median Ct Beads: 30.33 ± 7.59, Median Ct Qiagen 27.62 ± 7.56) (Quality control 1), measured by singleplex TaqMan qRT-PCR (individual values plotted in Figure S1A,B). Consistency of RNA extraction efficiency between samples was as expected, as measured by Cel-miR39-3p (Median Ct Beads: 23.81 ± 1.30, Median Ct Qiagen: 24.84 ± 5.25, Figure 2A). Serum/plasma levels of internal control hsa-miR30b-5p were also within the expected range (Median Ct Beads: 30.33 ± 7.59, Median Ct Qiagen: 27.62 ± 7.56, Figure 2B). The bead capture procedure displayed little variation with the spike-in control, whereas the Qiagen kit showed a consistent difference (~2 Ct) between control serum and plasma samples (Figure 2A). In the TGCT patient serum samples, again, the bead-based protocol resulted in little variation with the spike-in, where the Qiagen kit showed more differences in isolation efficiency (up to ~5Cts). Both methods showed little variation (or differences) relating to the endogenous hsa-miR30b-5p levels (Figure 2B). Due to differences in inputs (200 µL for Qiagen and 50 µL for beads) and elutions (50 µL Qiagen and 100 µL beads), a difference of 1Ct, in favor of the Qiagen kit, was expected. However, when comparing the Ct values for hsa-miR30b-5p for the Qiagen isolation and bead capture, the Qiagen kit overall showed a more efficient recovery of hsa-miR30b-5p (*p* < 0.0001), where the bead-capture showed a more efficient recovery of Cel-miR39-3p (*p* = 0.0002). In summary, little difference was observed in isolation efficiency between serum and plasma when using a bead-capture isolation protocol, where the Qiagen kit probably had a slightly better detection limit (Figure 2C).

### 3.2. Qiagen Kit MiRNA Isolation Has a Higher Detection, Whereas Both Kit and Bead Isolation Display Similar Results between Serum- and Plasma-Isolated Samples

**Figure 3:**
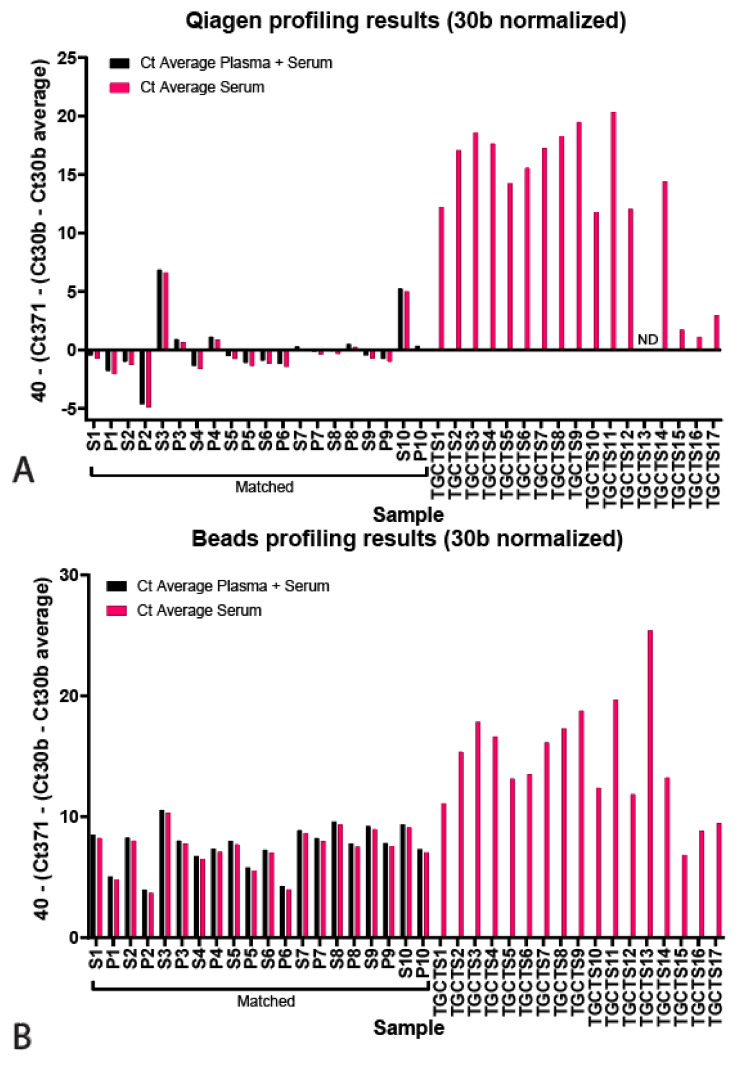

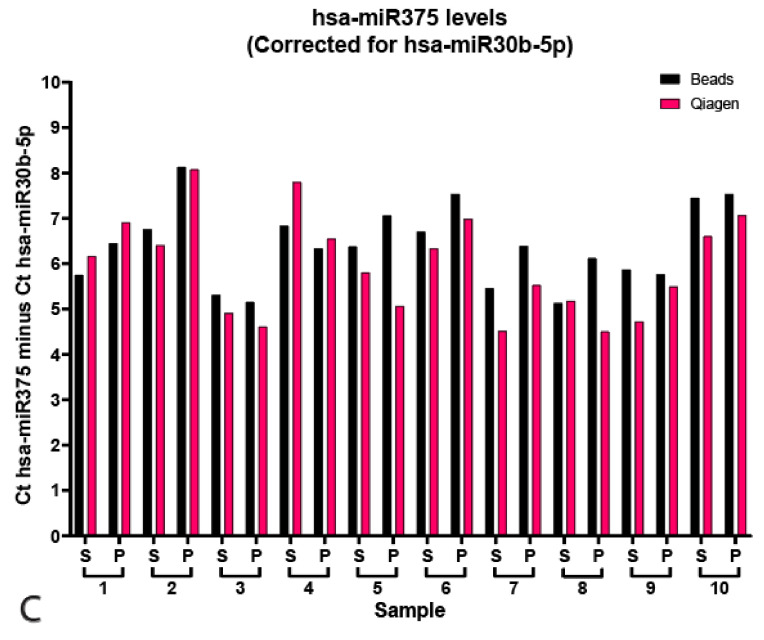
Targeted miR profiling. (**A**) Qiagen isolated levels of hsa-miR371a-3p levels plotted as 40—(Ct371—(Ct30b—Ct30b average) for plasma and serum (black) or serum only (pink). (**B**) Bead-capture isolated levels of hsa-371a-3p levels plotted as 40—(Ct371—(Ct30b—Ct30b average) for plasma and serum (black) or serum only (pink). (**C**) Levels of hsa-miR375 plotted as Ct hsa-miR375—hsa-miR30b for bead-capture (black) or Qiagen kit (pink) isolated samples. ΔCt Qiagen vs. Beads 0.65 ± 0.50, *p* = 0.16), ΔCt serum vs. plasma Qiagen 0.83 ± 0.40, *p* = 0.64, ΔCt serum vs. plasma Beads 0.63 ± 0.43, *p* = 0.22.

Next, we profiled the serum and plasma samples for hsa-miR371a-3p, normalized for hsa-miR30b-5p and displayed as 40—normalized Ct. The results are displayed for the Qiagen kit and bead-capture in Figure 3A,B, respectively (*p* values in Table 2, raw data and correction in Tables S1 and S2). Black bars represent samples that were corrected for the average hsa-miR30b-5p Cts among combined serum and plasma samples, whereas pink bars represent data corrected for the average Ct of only serum samples (because TGCTS samples were only serum-derived, these were only corrected for serum averages). The Qiagen kit resulted in less low-level detection and showed comparable results between serum and plasma samples, except for S3 and S10, showing some low-levels of the GCT miRNA (Ct of ~33 and 35, respectively) Furthermore, hsa-miR371a-3p could be detected in all TGCT sera, except in the case of TGCTS13, being excluded from the analysis due to high viscosity after protein precipitation (Qiagen kit only), and TGCTS 15–17, which were pure seminomas known to express low levels of hsa-miR371a-3p. Notably, TGCTS 15, 16 and 17 were derived from patients that had normal levels of the standard biomarkers AFP, bHCG and LDH and had tumors <2 mm (Table 3). The bead-capture procedure showed more low-level detection, e.g., the control sera displayed some levels (Ct of ~30–35) of the TGCT-specific miRNA. All tested TGCTS samples were positive for hsa-miR371a-3p, albeit lower levels were detected in TGCTS 15–17, again, due to these samples being known to have low levels of biomarkers (Table 2). Correction for endogenous control hsa-miR20a-5p produced similar results (Figure S2 and Tables S3 and S4). Finally, we also measured the levels of hsa-miR375 in 10 matched serum and plasma samples of healthy control samples and corrected for endogenous hsa-miR30b-3p (Ct hsa-miR375–Ct hsa-miR30b-5p, Figure 3C and Table S5). We present no differences between the Qiagen kit or bead-capture-based miRNA isolation (ΔCt Qiagen vs. Beads 0.65 ± 0.50, *p* = 0.16). Furthermore, we did not find differences between serum or plasma samples (ΔCt serum vs. plasma Qiagen 0.83 ± 0.40, *p* = 0.64, ΔCt serum vs. plasma Beads 0.63 ± 0.43, *p* = 0.22, Raw data presented in Table S5).

### 3.3. Input Titration Suggests Bead-Capture to Be Superior in Low-Volume Ranges

**Figure 4:**
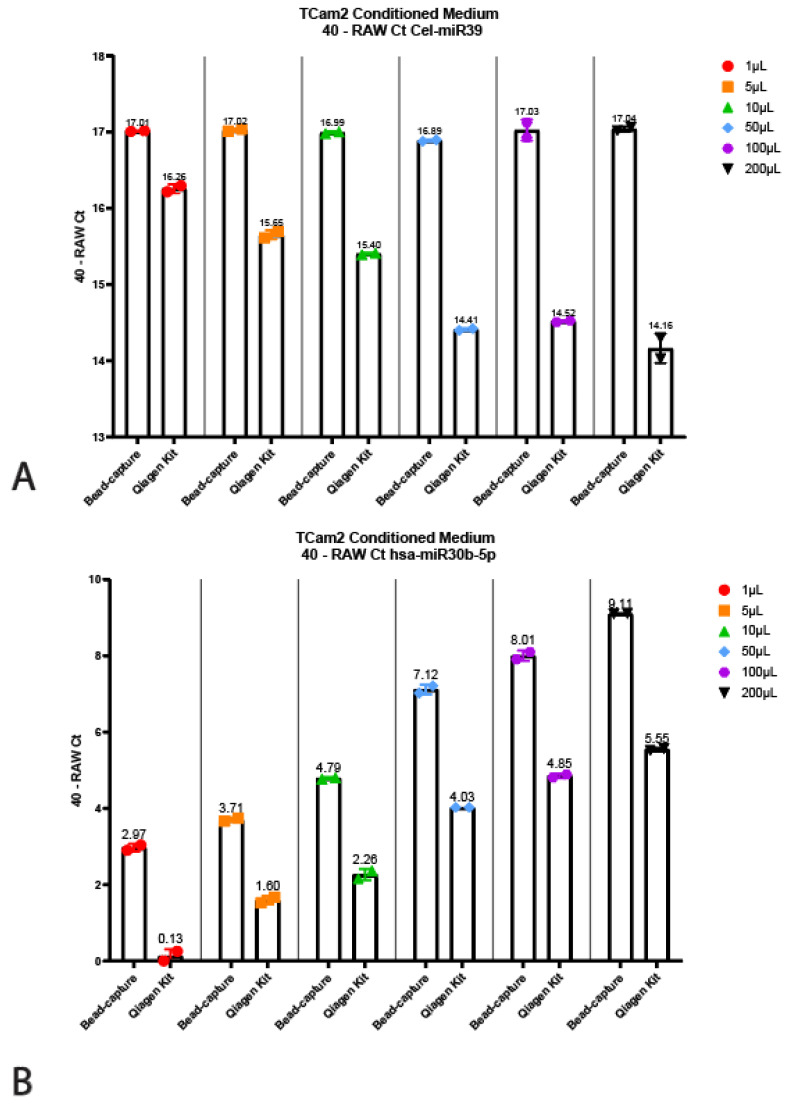

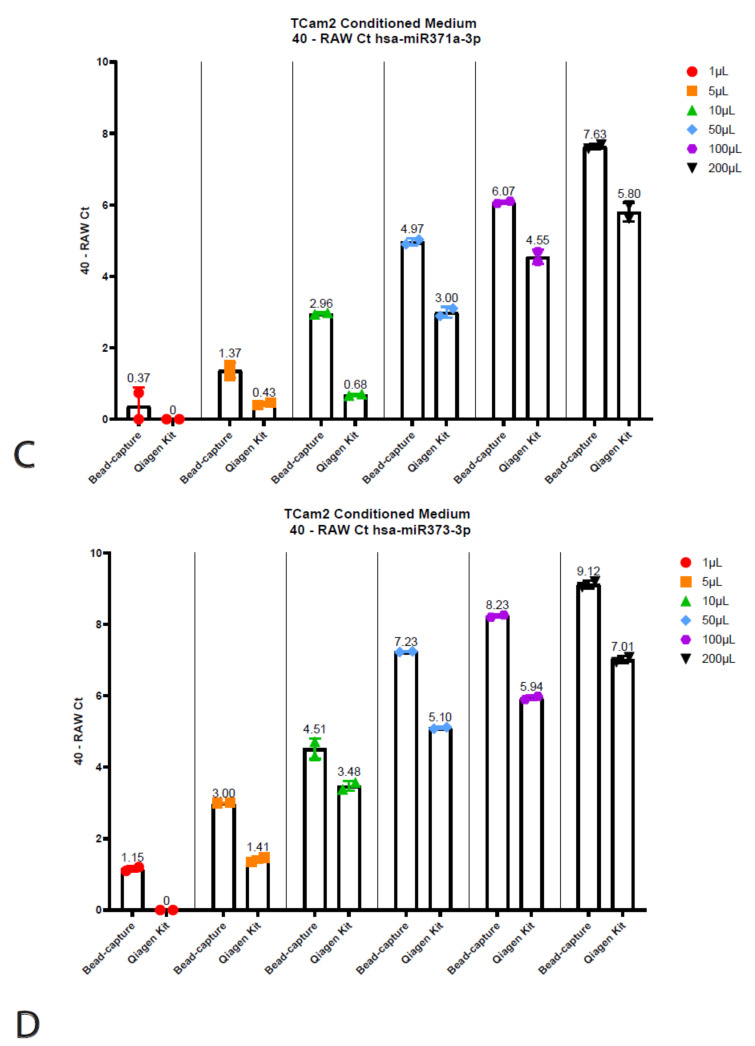
Titration using TCam2 conditioned medium. Displayed are the 40—Raw Ct values isolated using the bead-capture or Qiagen kit for Cel-miR39 (**A**), hsa-miR30b-5p (**B**), hsa-miR371a-3p (**C**) and hsa-miR373-3p (**D**). A 40 minus transformation was performed to improve the visual interpretation of data (high bars mean more target detected).

The Qiagen kit and the beads required different input volumes (200 µL vs. 50 µL, respectively, see above); therefore, we wanted to interrogate whether this had any influence on the quantitative output or detected miRNA levels. To test this, we titrated TCam-2 (TGCT cell line) conditioned medium into various volumes ranging from 1 µL to 200 µL, all diluted in PBS up to 200 µL (Figure 4). Note that conditioned medium does not give a full representation of serum and/or plasma; it can, however, faithfully demonstrate the reproducibility of the technique. Surprisingly, the 40 minus RAW Cts for the Cel-miR39 for the Qiagen kit decreased as the input increased (Figure 4A). However, when using bead-capture isolation, no differences in miRNA detection could be observed when using different input volumes. When using different input volumes to detect endogenous miRNA levels (hsa-miR30b, 371a-3p and 373), the bead-based method was shown to be superior (Figure 3B–D). Even when using 1 µL (instead of the recommended 50 µL) for bead-capture isolation, we were able to isolate detectable levels of all three miRNAs (Figure 4). As expected, increasing the amount of input also increased detection levels. Using a 1 µL starting volume resulted in low (hsa-miR30b-5p) or undetectable miRNA (hsa-miR371a-3p and 373) levels when using the Qiagen kit, increasing the detection levels as the input volume increased.

## 4. Discussion

Ever since the study published by Lawrie and colleagues in 2008, miRNAs in bodily fluids have been an interesting target for oncogenic biomarker detection [24]. With the discovery of the miR371a-3 cluster and its specific expression in (T)GCTs [25,26], miRNAs have become a suggested cornerstone in (T)GCT detection and monitoring [6,7,9,10,11]. Many studies have investigated the effects of sample handling on the outcome of biomarker identification studies [4,5,15,16,17,18,19,20,21]. Not only have these studies reported (minor) differences in sample handling, but different groups all over the world use various techniques, protocols (i.e., Bead-capture vs. Qiagen kit) and starting materials (i.e., serum vs. plasma). Where most groups report similar results, especially regarding the fidelity of the miR371a-3 cluster in (T)GCT detection [6,7,9,10,11], some discrepancies have arisen in the field regarding biomarkers to specifically detect residual teratoma [8,12,13,14]. These discrepancies have been suggested by several authors [12,14] to be linked to differences between starting material, i.e., serum vs. plasma, and isolation protocol, i.e., Qiagen kit vs. bead-capture. Here, we report a comprehensive analysis of two relevant aspects in the field: (1) the isolation technique: ThermoFisher’s TaqMan anti-miRNA bead-capture procedure (~370 miRNAs) vs. the miRNeasy Serum/Plasma Advanced Kit Qiagen extraction kit (cell-free total RNA purification), two of the most commonly used isolation methods; and (2) serum vs. plasma as starting materials [27]. For this study, we used ThermoFisher’s RT-primers and assays (Materials and Methods) to detect the miRNAs because these assays have been used by many groups in the field, allowing us to faithfully compare the isolation method and starting material independent of detection [27]. First, we used spike-in Cel-miR39-3p spike-in and endogenous hsa-miR30b-5p as controls to validate to isolation efficiency. We identified an overall lower variation in detection levels and better recovery with the bead-based method (Median Ct Beads: 23.81 ± 1.30, Median Ct Qiagen: 24.84 ± 5.25, *p* = 0.0002) for Cel-miR39-3p. Furthermore, we found an overall lower detection limit but equal variation for endogenous miR30b-5p using the Qiagen kit isolation (Median Ct Beads: 30.33 ± 7.59, Median Ct Qiagen: 27.62 ± 7.56, *p* < 0.0001), partly accounting for the difference in input volume and total vs. targeted RNA isolation. We compared the differences between serum and plasma using both the Qiagen and bead-based (mi)RNA isolation (results normalized for both endogenous hsa-miR30b and 20a). We observed no differences between serum and plasma in hsa-miR371a-3p levels when isolating miRNAs using the Qiagen kit, whereas the bead-capture performed better when using serum samples (*p* = 0.0039). There was no difference in the detection of hsa-miR371a-3p in TGCT patient serum samples between the Qiagen kit isolation of the bead-capture (*p* = 0.56), whereas bead-capture did detect significantly higher levels of hsa-miR371a-3p in healthy donors (*p* < 0.0001). Both the Qiagen kit and the bead-capture isolation resulted in the detection of significantly higher levels of hsa-miR371a-3p (*p* < 0.0001 and *p* = 0.0003, respectively). The majority of miR371a-3p-related studies are performed on serum samples. However, as concluded in the recent systematic review on the use of this microRNA as a biomarker for TGCT [27], similar overall results have been obtained when serum or plasma were used as starting materials. This is amongst other analyses based on the matched serum and plasma samples of 50 healthy males, showing similar results using the ampTSmiR assay [7,28]. These results show consistent differences between the normalizer (hsa-miR30b-5p) and the target hsa-miR371a-3p, being higher and lower in plasma versus serum, respectively. Therefore, it was concluded that mixed series of both serum as well as plasma will be problematic regarding normalization, and as a result, interpretation. The consistent differences between plasma and serum samples reported is confirmed independently in the study presented here, being independent of the isolation technique applied. Moreover, 25% of the studies included in the forementioned systematic review [27] were based on the bead-capture-based method, whereas the others used a total RNA isolation method, demonstrating the relevance of this comparative analysis. Even when we compared the levels of hsa-miR375 between the serum and plasma of healthy males, we found no difference between serum and plasma (ΔCt serum vs. plasma Qiagen 0.83 ± 0.40, *p* = 0.64, ΔCt serum vs. plasma Beads 0.63 ± 0.43, *p* = 0.22). Furthermore, because we observed clear detectable levels of hsa-miR375 in 10 healthy males (both serum and plasma), we support the findings of Lafin and colleagues, showing that hsa-miR375 is not suitable as a teratoma biomarker at present [13]. Thirdly, because we did not observe any differences between serum and plasma with either hsa-miR371a-3p or hsa-miR375 when using the Qiagen kit (*p* = 0.18), we conclude that this therefore cannot explain reported inconsistencies regarding hsa-miR375 as a teratoma marker as well, also supported by findings in a recent systematic review [12,13,14,27]. Finally, because the Qiagen kit and bead-capture require different input volumes, we used TCam-2 conditioned medium to detect the differences in miRNA levels between different input volumes. We report an overall lower detection limit for the Qiagen kit, possibly related to total RNA extraction versus targeted extraction. However, when using increasing volumes of Cel-miR39-3p, we found lower levels with the Qiagen kit. In other words, adding more input with the same amount of spike-in detected less of the miRNA when isolating the samples using the Qiagen kit. This is likely explained by the loss of spike-in miRNA during the precipitation step.

## 5. Conclusions

We conclude that in low volume ranges, the bead-capture method is therefore superior and more useful for studies with young patients or mice where less starting volume is available. In summary, the Qiagen kit is the preference compared to the bead-based approach for expected low-expressed miRNAs. However, when limited sample volume is available, the bead-capture method outperforms the Qiagen kit. These results will aid future studies to determine the optimal isolation method for miRNA detection both using serum and plasma. 

## Figures and Tables

**Table 1:** Sample codes. Corresponding samples linked to codes used in figures.

#	Code	Serum/Plasma	Graph Annotation
1	S-267	normal serum	S1
2	P-299	normal plasma	P1
3	S-306	normal serum	S2
4	P-326	normal plasma	P2
5	S-254	normal serum	S3
6	P-279	normal plasma	P3
7	S-255	normal serum	S4
8	P-289	normal plasma	P4
9	S-310	normal serum	S5
10	P-331	normal plasma	P5
11	S-261	normal serum	S6
12	P-277	normal plasma	P6
13	S-263	normal serum	S7
14	P-293	normal plasma	P7
15	S-270	normal serum	S8
16	P-285	normal plasma	P8
17	S-265	normal serum	S9
18	P-280	normal plasma	P9
19	S-268	normal serum	S10
20	P-300	normal plasma	P10
21	L10-156	serum TGCT (YST)	TGCTS1
22	L11-107	serum TGCT (YST)	TGCTS2
23	L11-160	serum TGCT (mixed NS)	TGCTS3
24	L12-360	serum TGCT (mixed NS)	TGCTS4
25	L12-067	serum TGCT (mixed NS)	TGCTS5
26	L13-035	serum TGCT (EC)	TGCTS6
27	L13-109	serum TGCT (mixed NS)	TGCTS7
28	L13-121	serum TGCT (mixed NS)	TGCTS8
29	L13-138	serum TGCT (EC)	TGCTS9
30	L12-187	serum TGCT (mixed NS)	TGCTS10
31	L12-026	serum TGCT (mixed NS)	TGCTS11
32	L17-220	serum TGCT (mixed NS)	TGCTS12
33	L18-141	serum TGCT (SE)	TGCTS13
34	L14-254	serum TGCT (mixed NS)	TGCTS14
35	L15-193	serum TGCT (SE)	TGCTS15
36	L15-402	serum TGCT (SE)	TGCTS16
37	L18-137	serum TGCT (mixed NS)	TGCTS17

Abbreviations: SE: seminoma, NS: non-seminoma, EC: embryonal carcinoma, YST: yolk-sac tumor.

**Table 2:** *p*-values of hsa-miR371a-3p detection in healthy control and TGCT patient samples corrected for hsa-miR30b-5p.

Comparison	*p*-Value
Qiagen kit healthy donor serum vs. plasma	0.18
Qiagen kit healthy serum vs. TGCT serum	<0.0001
Bead-capture healthy donor serum vs. plasma	0.0039
Bead-capture healthy serum vs. TGCT serum	0.0003
Healthy serum + plasma samples Qiagen vs. Beads	<0.0001
Healthy serum samples Qiagen vs. Beads	<0.0001
Healthy plasma samples Qiagen vs. Beads	<0.0001
TGCT serum Qiagen vs. Beads	0.56

**Table 3:** Serum marker TGCT patients. Serum marker levels of classical GCT serum markers AFP, b-HCG and LDH. Cells marked with red indicate clinically elevated (above threshold) serum levels of these markers.

		Pre-	Orchiectomy	Markers
	Sample-nr.	AFP ug/L	b-HCG IU/L	LDH U/L
1	21	856	<0.1	190
2	22	1349	<0.1	237
3	23	669	829	275
4	24	2123	2698	583
5	25	90	<0.2	98
6	26	1	6	354
7	27	40	400	166
8	28	175	4,5	195
9	29	35	65	275
10	30	29	1268	365
11	31	1.3	0.1	712
12	32	465	210	167
13	33	1	<1.0	1610
14	34	24	33	219
15	35	0.9	0.8	255
16	36	1.4	0.3	237
17	37	5	4.3	181

## Data Availability

The data presented in this study are available on request from the corresponding author. The data are not publicly available due to presence of patient data from different institutes and the pending of a patent.

## Supplementary Materials

The following are available online at https://www.mdpi.com/article/10.3390/cancers13174260/s1, Figure S1: Quality control values of Spike-in Cel-miR39-3p (A) and has-miR30b-5p (B). Figure S2: Qiagen kit (A) and bead-capture (B) isolated levels of hsa-miR371a-3p plotted as 40 - (Ct371 - (Ct20a - Ct20a average). Table S1: Raw data and corrections for hsa-miR371a-3p corrected for hsa-miR30b-5p, extracted using the bead-capture technique. Table S2: Raw data and corrections for hsa-miR371a-3p corrected for hsa-miR30b-5p extracted using the Qiagen kit. Table S3: Raw data and corrections for hsa-miR371a-3p corrected for hsa-miR20a-5p extracted using the bead-capture technique. Table S4: Raw data and corrections for hsa-miR371a-3p corrected for hsa-miR20a-5p extracted using the Qiagen kit. Table S5: Raw Ct values for hsa-miR375.
